# Influence of Growth Regulators on Callogenesis and Somatic Embryo Development in Date Palm (*Phoenix dactylifera* L.) Sahelian Cultivars

**DOI:** 10.1100/2012/837395

**Published:** 2012-05-02

**Authors:** Djibril Sané, Frédérique Aberlenc-Bertossi, Léopold Ibrahima Djitiningo Diatta, Badara Guèye, Abdourahman Daher, Maurice Sagna, Yves Duval, Alain Borgel

**Affiliations:** ^1^Département de Biologie Végétale, Université Cheikh Anta Diop, BP 5005, Dakar-Fann, Senegal; ^2^Institut de Recherche pour le Développement, IRD, BP 64501, 34394 Montpellier Cedex 5, France; ^3^Institut des sciences de la vie, Centre d'Etudes et de Recherche de Djibouti (BP 486), BP 5005, Dakar-Fann, Djibouti

## Abstract

This study provides a physiological analysis of somatic embryogenesis in four elite cultivars of date palms: Ahmar, Amsekhsi, Tijib, and Amaside, from the initial callogenesis to establishment and proliferation of embryogenic suspension cultures. Somatic embryos development and in vitro plants rooting were also studied. For each step, auxins and cytokinins concentrations were optimised. The primary callogenesis from leaf explants of seedlings appeared highly dependent on genotype. Ahmar (80%) and Amsekhsi (76%) appeared highly callogenic, whereas Tijib (10%) and Amaside (2%) produced low amounts of calluses. 2,4-Dichlorophenoxyacetic acid appeared favorable to the induction of primary callogenesis and its effect was enhanced by the addition of benzyl adenine or adenine sulfate. Secondary friable calli obtained from chopped granular calli were used to initiate embryogenic cell suspensions in media supplied with 2,4-dichlorophenoxyacetic acid. Suspension cultures showed a growth rate of fourfold after four subcultures in presence of 2,4-dichlorophenoxyacetic acid 2 mg/L. Our results showed that a seven-day transitory treatment with benzyl adenine 0,5 mg/L was necessary to optimize embryos development. Naphthalene acetic acid induced the development of primary orthogravitropic roots during embryos germination. The comparison with cytofluorometry of nuclear DNA amounts showed no significant difference in ploidy level between regenerated plants and seedlings.

## 1. Introduction

The date palm (*Phœnix dactylifera* L.) is a dioecious perennial species of the Arecaceae family, adapted to arid zones where it plays an economic role thanks to the production of dates. Date palm plantations which often constitute the framework of oases mainly encountered in North Africa, Middle East, and Sahel are traditionally propagated by seeds or offshoots. To ensure the renewal and extension of date palm groves, *in vitro* micropropagation techniques have been developed from zygotic embryos, axillary buds, and immature leaves [1–4]. Date palm micropropagation through somatic embryogenesis has been previously reported [5, 6]. The use of embryogenic suspension cultures improved the yields of the regeneration processes and allowed large-scale propagation of several date palm cultivars [5, 7–9]. Recently, regeneration through somatic embryogenesis from embryogenic suspensions culture was obtained for the first time for the Sahelian cultivar Amsekhsi [10].

However, the efficiency of various somatic embryogenesis protocols described for date palm depends on the cultivars, some of them being recalcitrant to *in vitro* culture [9, 11]. One of the major bottlenecks in regeneration procedures is the production of primary calli. Exogenous auxins and cytokinins are the main plant growth regulators (PGRs) involved in the control of cell division and differentiation [12]. The role of these PGRs in the regeneration performance of date palm has been previously described [8–10].

It is therefore of importance to optimise the somatic embryogenesis conditions in order to extend the regeneration protocols to genotypes which are specifically adapted to the sahelian edaphoclimatic conditions. In the present study, the effect of various PGRs, particularly of the auxins, 2,4-dichlorophenoxyacetic acid (2,4-D) and naphthalene acetic acid (NAA), and of the cytokines, benzyl adenine (BA) and adenine sulfate on the callogenesis of four sahelian cultivars was explored. The hormonal conditions for the proliferation of embryogenic cell suspensions, the development of somatic embryos, and the rooting of vitroplants were also investigated. The nuclear DNA content of regenerated plantlets was controlled by flow cytometry.

## 2. Material and Methods

### 2.1. Plant Material and Preparation of Explants

Our study was implemented from seeds obtained from four cultivars, namely: Ahmar, Amsekhsi, Tijib, and Amaside harvested in plantations in the Atar region (Mauritania).

The seeds were sterilised with 96% H_2_SO_4_ for 10 min then rinsed with sterile distilled water. They were then soaked in sterile water for 24 h before being transferred onto glass tubes (25 × 150 mm) containing 20 mL of agar (Difco Agar) (8 g/L). After one month of culture in a controlled culture room with a 12 h/12 h photoperiod and a light flow of 80 *μ*E·s^−1^ · m^−^², at 27° ± 0.2°C, the seedlings where dissected. Young leaves were cut into segments of 1 cm in length. All the explants were transferred for callus induction on various growth regulators concentrations. 

### 2.2. Primary and Secondary Callogenesis

For each of the 4 studied cultivars, 48 segments were used by type of explant (shoots or roots) and by medium condition. The explants were placed on a basic medium composed of Murashige and Skoog solution [13], FeEDTA, Morel and Wetmore vitamins [14], biotin (0.01 mg/L), sodium ascorbate (100 mg/L), and myoinositol (100 mg/L) [10]. This medium was supplied with sucrose (30 g/L), agar (Difco Agar) (8 g/L), and increasing concentrations of 2,4-D (1, 2, 4, 8, or 16 mg/L) or NAA (2 or 4 mg/L) combined or not with BA (1 mg/L) or adenine sulfate (40 mg/L). The pH was adjusted to 5.5. The effect of the hormonal composition was evaluated by counting of the calluses obtained after 2 months of culture in the dark at 27 ± 0.2°C.

The primary calluses were chopped with a scalpel according to Teixeira et al. [15] and transferred on the basic medium supplied with 2,4-D (2 mg/L). After one month, secondary calluses were used for the installation of embryogenic suspension cultures. They were placed in Erlenmeyers flasks containing 50 mL liquid medium of the same composition without agar and cultivated on an orbital shaker at 90 rpm in light (80 *μ*E·s^−1^·m^−^²) with a photoperiod of 12 h/12 h at 27 ± 0.2°C. 

### 2.3. Establishment of Embryogenic Suspension Cultures

The protocol used is adapted from that described in the oil palm [16]. Each month, 300 mg fresh weight (FW) of cell suspensions was transferred onto a liquid medium containing the basic medium supplemented with 20 g/L of glucose. The effect of 2,4-D, used at 2 mg L^−1^ without activated charcoal or at 50, 75 and 100 mg·L^−1^ with activated charcoal (1 g/L) was evaluated by monitoring growth rates of the suspension cultures. For each condition, FW of proembryogenic masses (PEMs) was measured monthly during four subcultures (five repetitions).

### 2.4. Development of Somatic Embryos

In order to produce somatic embryos, the suspensions were cultivated for one month in a liquid medium containing the basic medium without hormone. Suspensions were then filtered through a double nylon mesh (1 and 2 mm). PEMs (50 mg FW) were transferred for one week onto a filter paper in a 9 cm diameter Petri dish containing 20 mL of basic medium enriched with sucrose 60 g L^−1^ and containing 0, 0,5; 1; 1,5, or 2 mg/L of BA and gelified with agar at 8 g/L. For each culture condition, 5 Petri dishes were used. The filter papers and the cultures were then transferred on the same medium deprived of hormone and subcultured weekly during five weeks. The effect of the application of various BA concentrations on the evolution of PEMs biomass and the growth (number and size) of somatic embryos was then evaluated.

### 2.5. Germination of Somatic Embryos and Rooting

Developed somatic embryos (length 10 to 11 mm) were transferred for germination in individual glass tubes (25 × 150 mm) on Murashige and Skoog medium [13], with or without NAA (1 mg/L). For each conditions, 48 embryos were used. The effect of NAA on the morphology of the root system was evaluated by the number and the length of roots produced after 4 weeks of culture. The rooted plantlets were then transferred in the greenhouse.

### 2.6. Cytofluorimetric Analysis of Ploïdy Level

The genetic stability of the clones was estimated by measuring the nuclear DNA content in *in vitro* plantlets and seedlings. The method developed by flow cytometry was performed using an argon laser cytometer (15 mW) (FAC-Scan, Becton Dickinson) with emission at 488 nm [17]. The extraction of interphasic nuclei was carried out by manual chopping of 30 mg of the sample in 1.5 mL of lysing buffer [18, 19]. Nuclei were stained with 330 *μ*g·mL^−1^ propidium iodide during 5 min on ice. Each sample was analyzed on the basis of 5 independent repetitions. For each analysis, 3,000 nuclei were measured. Fluorescent latex beads (2 *μ*m diameter) were used as internal standard. The estimation of the nuclear DNA content was carried out from foliar segments of same developmental stages harvested on *in vitro* plantlets from three clones from the cultivar Ahmar (Ahm A1, Ahm A8, and Ahm A14-F) and three clones of Amsekhsi (Amse A56, Amse A57, and Amse A72). Foliar segments were obtained from the germination of seeds of the same cultivars. The size of the date palm genome was compared to that of the reference rice variety Nippon Bar (2C = 1.00 pg) [17].

### 2.7. Statistical Analysis

For each stage of somatic embryogenesis, the experimental design was randomized. Statistical analyses were carried out by using the Linear Model ANOVA/MANOVA of STATISTICA (analysis software system dated), version 6. StatSoft, Inc. (2001). The treatments were discriminated by multiple mean comparison of after-variance analysis followed by Newman and Keuls test (threshold 5%) or by Pearson's Chi-squared test.

## 3. Results

### 3.1. Callogenic Capacity Depends on the Cultivars and the PGR Composition of the Medium

**Figure 1:**
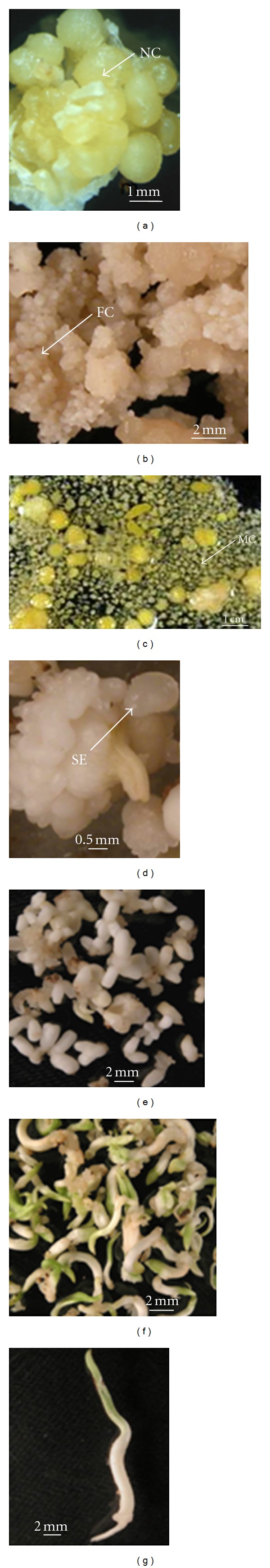
Callogenesis and development of date palm somatic embryos. (a) compact nodular primary callus obtained from foliar explants after 2 months on 2,4-D (2 mg l^−1^) containing medium; (b) friable secondary callus obtained 1 month after chopping of the primary callus; (c) suspension culture; (d) somatic embryos of stage I, (e) stage, II and III (f), respectively, after 2, 4, and 6 weeks of culture on PGR-free medium; (g) germinated somatic embryo. NC: Nodular callus, FC: Friable callus, MC: microcallus, SE: somatic embryo.

The effect of PGRs (auxins and cytokines) on the callogenesis capacity of foliar and root explants was evaluated for the four date palm cultivars studied. The observations carried out after 8 weeks of culture revealed the elongation of all the roots segments placed in culture. However, no callus formation was observed on root explants, whatever the hormonal combination tested. On the other hand, a morphogenetic activity characterized by the proliferation of compact granular callus was observed on foliar explants after 4 weeks of culture in 2,4-D-containing mediums. From 2 to 3 mm in diameter, these callus developed and reached a 6-7 mm size after 8 weeks (Figure 1(a)).

**Figure 2:**
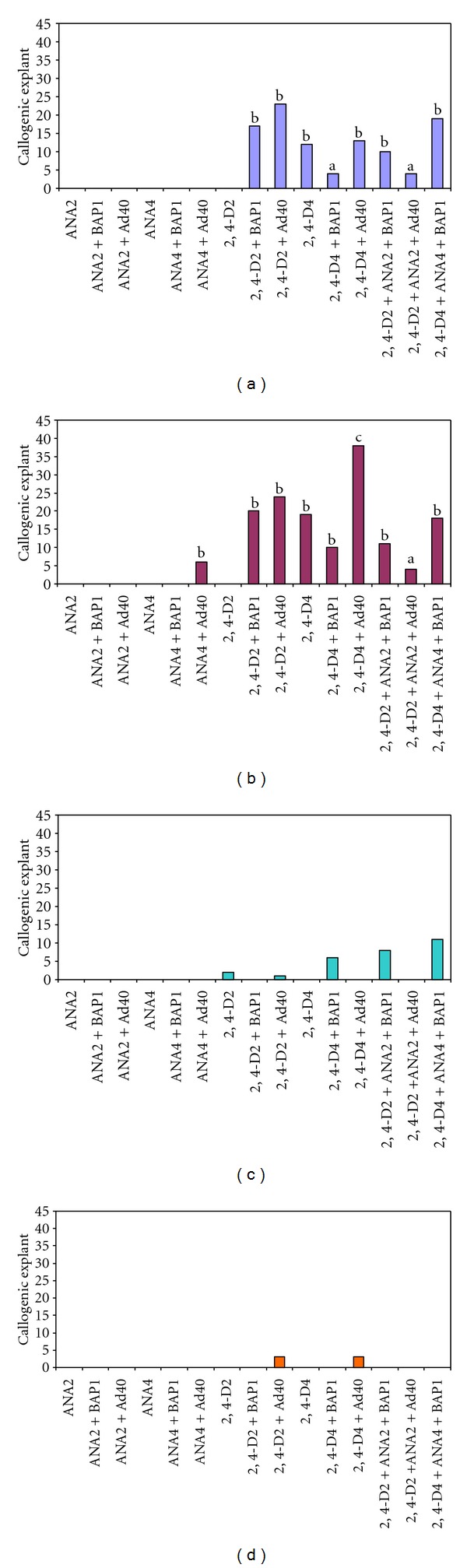
Effect of plant growth regulators (PGRs) in mg/L on the induction of callogenesis after 60 days of culture for cultivars Ahmar (a), Amsekshi (b), Tijib (c) and Amaside (d). Means were calculated from 48 explants per condition. Letters indicate significant differences according to Newman and Keuls test at the level of 5%.

The effect of auxin on callogenesis from foliar explants was studied for the four sahelian cultivars (Figure 2). Two of the 4 cultivars (Ahmar and Amsekhsi) were found to be very callogenic (resp. 80% and 76%), whereas Tijib and Amaside exhibited very low callusing rates (resp. 10% and 2%). For the two most callogenic cultivars, almost no callus formation was observed in the presence of NAA only or combined with BA or adenine sulfate (Figures 2(a) and 2(b)). Callogenesis was mostly observed on 2,4-D-enriched medium. The induction of primary callogenesis required 2,4-D concentrations ranging from 2 to 4 mg/L. The addition of BA or adenine induced higher callus frequencies (Figures 2(a) and 2(b)).

When globular and compact primary callus were chopped, they gave rise after between 6 to 8 weeks of culture, to friable granular secondary callus which will be used for the initiation and the installation of embryogenic suspension cultures (Figure 1(b)).

### 3.2. Proliferation of the Embryogenic Suspension Cultures

**Figure 3:**
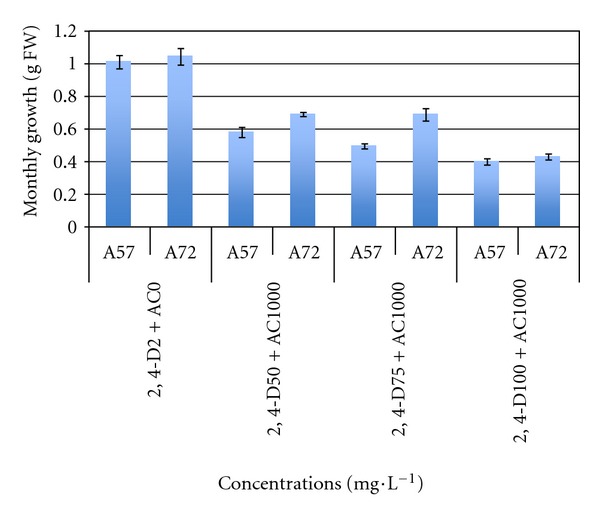
Effect of 2,4-D (2 mg/L) without activated charcoal (AC) or at 50, 75, and 100 mg/L with AC (1000 mg/L) on growth rates of suspension cultures from two different Ahmar cultivars lines. Averages values were calculated from 5 repetitions per condition of medium; bars indicate confidence interval at 95% threshold.

The cultures (consisting in microcallus suspensions) were established after two subcultures in liquid media enriched with 2,4-D with or without active charcoal (Figure 1(c)). Growth rates were evaluated during 4 successive subcultures for two clones of the Ahmar cultivar, namely, A57 and A72.  Figure 3 shows that growth rates were variable depending on both the clone and the 2,4-D level. Variance analysis (95% threshold) showed very significant differences in growth rates between the 2 clones (*F* (3, 32) = 40.748; *P* = 0.000). In the presence of activated charcoal, any increase in the amount of 2,4-D beyond 50 mg/L significantly decreased growth rates. However, whatever the clone considered, the 2 mg/L concentration of 2,4-D was the most favorable for cellular proliferation, allowing monthly multiplication rates of fourfold.

### 3.3. Development of Somatic Embryos

The development of somatic embryos was obtained after a one-month cultivation period on a PGR-free medium, followed by plating on semisolid medium. The cultures were maintained on BA-containing medium for 1 week then on PGR-free medium for five weeks. Somatic embryos of stage I (length 1 to 1.5 mm, 1 mm diameter) epidermized and of ovoid shape were observed as of the 2nd week of culture (Figure 1(d)). The development of embryos was optimized by a one-week treatment with BA 0.5 mg/L (on average 29 to 30 embryos from 40 mg of suspension culture) (Table 1). An increase in cytokinin concentration induced a significant decrease of the number of individualized embryos and an increase in the number of vitrified embryos (*F* (12, 100.83) = 6.6151; *P* = 0.000). The development of somatic embryos of stage II (length 4 to 5 mm, 1.5 mm diameter) was obtained between the 3rd and the 4th week of culture (Figure 1(e)). After 6 weeks of culture, these embryos developed in chlorophyllous somatic embryos of stage III (length 10 to 11 mm, 1.7 to 2 mm diameter) (Figure 1(f)).

### 3.4. Germination of Somatic Embryos and Rooting of Vitroplants

The mature somatic embryos of stage III developed a shoot and a root and germinated with a rate of 82%. The morphology of roots produced after 4 weeks of culture depended on the NAA concentration (Table 2). In a PGR-free medium, plants produced numerous fine and plagiotropic roots, 1.4 to 2.3 cm long. In contrast, when embryos were cultivated with 1 mg/L NAA, the plants developed a vigorous and orthotropic root (3.3 to 4.9 cm long) whose morphology was similar to that obtained during the germination of seedlings (Figure 1(g)).

### 3.5. Nuclear DNA Content

**Figure 4:**
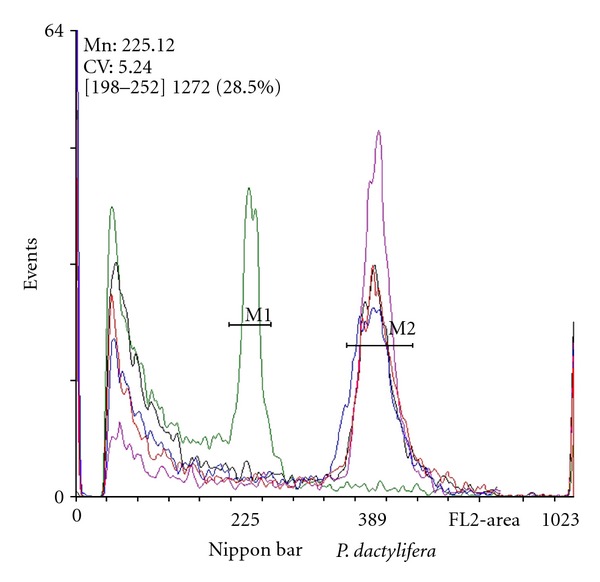
Flow cytometry analysis of nuclear DNA from leaves of rice (Nippon bar) seedlings (green), date palm cv. Ahmar seedlings (violet), and date palm cv. Ahmar somatic embryo-derived plantlets (red, black, and blue).

Measurements of nuclear DNA content were standardized using leaves of Nippon Bar rice variety as an internal standard (2C = 1 pg DNA by nucleus). The size of the genome of *Phoenix dactylifera *as estimated on seedling leaves from the cultivars Ahmar and Amsekhsi was 2C = 1.74 pg/nucleus and 2C = 1.73 pg/nucleus (resp.). No significant difference was found between the values obtained from seedlings and somatic embryo-derived plantlets (*F* = 0.507; *P* = 0.82) (Table 3). The cytofluorimetric analysis revealed that all the regenerated clonal offsprings were diploïd (Figure 4) as one peak at 2C DNA nuclear content was observed. No 3C, 4C, 6C, or 8C peak indicating changes in ploïdy level could be found. 

## 4. Discussion

The regeneration process developed for the sahelian date palm cultivars allowed the production of somatic embryoderived plantlets through indirect embryogenesis involving two callogenesis stages and embryogenic suspensions culture. Each step was optimised by using various PGR concentrations.

The aptitude for primary callogenesis appeared to be strongly dependent on the explant nature, the genotype, and the growth regulators used. The foliar segments from young date palm seedlings was found to be the optimal explant for callus induction compared to root tissues. For the four cultivars under study, primary callogenesis led to the formation of globular compact calluses similar to those described in *Elaeis guineensis* [15] and *Phoenix canariensis* [6].

The callogenic capacity was found to be more than seven times higher for the cultivars Ahmar and Amsekhsi than for the cultivars Tijib and Amaside. At the date palm was found that the genotype is one of the most important factors in the induction of primary callus [11]. Similar observations were made on coconut [20]. The reason(s) of the recalcitrance to callogenesis of some genotypes still remain unknown. The hypothesis of a genetic inability to callus formation was proposed, respectively, in *Medicago sativa* and *Zea mays* [21, 22]. The identification of quantitative trait loci (QTLs) associated with callogenesis and embryogenesis has been undertaken using molecular markers obtained from *Arabidopsis thaliana* tissue culture cDNA libraries [23]. Genetic mapping of such markers would allow the selection of genotypes with high ability to tissue culture.

The phase of cell multiplication, related to the sensitivity to the 2,4-D of foliar tissues of date palm, occurs as of the 2nd week of culture [10] and leads to the formation of the primary calluses after the 4th week of culture. The callogenesis stage requires the use of elevated exogenous auxin levels in many species [12]. The effect of the 2,4-D during cellular dedifferentiation was strongly correlated with an increase in endogenous AIA in carrot tissues [24]. Indeed, results obtained in *Medicago sativa* showed that concentrations in endogenous AIA increased considerably during the first 3 days of culture in the presence of optimal concentrations of 2,4-D [25]. The accumulation of endogenous AIA in tissues under 2,4-D treatment would be at the origin of the totipotency of somatic cells in *Zea mays* and consequently of their capacity to be directed towards embryogenesis [26].

Recent advances in auxin biology have clarified the mode of action, signalling, and gene expression of this plant hormone [27, 28]. Auxin induces the expression of several genes including *Aux/IAA*, *GH3* (IAA-aminoacid conjugating enzyme), and glutathione *S*-transferase [12, 29]. *GH3* protein homologs were strongly induced in response to 2,4-D [30], suggesting that this auxinomimetic could use the signalling pathway, at least partly, of auxin response.

In this study, the high callogenesis rates obtained for Ahmar and Amsekhsi cultivars in the presence of the combinations of 2,4-D with BA or adenine sulphate, which may act as a precursor of natural cytokinin [31] stresses the importance of the auxin/cytokinin balance during the early steps of embryogenesis in date palm. The addition of BA in culture media already containing 2,4-D also improved callogenesis rates in *Acacia raddiana* [32]. The dedifferenciation of protoplasts obtained from foliar cells of alfalfa could also optimize varying the auxin/cytokinin balance [25]. Both auxins and cytokinins can trigger somatic cells to differentiate into embryogenic competent cells [12]. During the somatic-to-embryogenic transition in *Arabidopsis thaliana*, transcription factors such as *BABY BOOM* (*BBM*) and* LEAFY COTYLEDON 1 (LEC1) *were activated [23, 33]. Ectopic expression of* BBM* and *LEC1* was sufficient to induce somatic embryo development from vegetative cells [23, 33]. It would be very interesting to study the effect of PGRs on the expression of such a gene in palm leaf tissues in relation to callogenesis progression and rates.

Whatever the hormonal balance experimented in this study, both cultivars Tijib and Amaside were found to be weakly callogenic. The recalcitrance of such genotypes could be overcome by the use of other auxins and cytokinins that were reported to be efficient for the induction of callogenesis in several palm species. In the betelnut palm (*Areca catechu*), callogenesis was induced on medium containing Dicamba, an auxinomimetic PGR [34]. In *Phoenix canariensis*, callogenesis was induced from shoot tips with 2,4-D and 2iP (2-isopentenyl adenin) or with Picloram and kinetin [6]. The combination of 2,4-D and 2iP was also found efficient in inducing callus development in date palm [3, 35].

The granular secondary calli used for the initiation of suspensions were obtained by chopping primary calli. Two to four subcultures were necessary to establish suspensions culture in the presence of 2,4-D. These suspensions consisted of embryogenic clumps [10] whose multiplication rate reached threefold on 2,4-D (2 mg L^−1^) containing medium. To ensure the development of somatic embryos, it was necessary to modify the hormonal balance in favour of the cytokinins. The transitory application of BA (0,5 mg L^−1^) improved embryo development after only 5 weeks. The omission of 2,4-D from the culture medium followed by the addition of BA enhanced the differentiation of proembryos toward bipolar embryos in oil palm [16] and coconut [36].

Under our experimental conditions, the development and subsequent germination of embryos were carried out on gelified PGR-free media. The germination rates of stage III embryos reached 82%. The application of NAA (1 mg·L^−1^) favoured rooting as already shown for *P. canariensis* [6] and *P. dactylifera* [10]. NAA induced the formation of a primary orthogravitropic root comparable to that developed during *in vitro* germination of oil palm seeds [37]. This root system may be more efficient during the critical acclimatization phase of the plantlets.

The somatic embryogenesis process described here allowed the production of approximately 10,000 individualized embryos from 15 g of suspension culture. It constitutes a first step towards the development of large-scale regeneration protocols for sahelian date palm cultivars. Our results illustrate the importance of PGR concentrations and of the balance between auxins and cytokinins in the optimisation of regeneration protocols. For each genotype the identification of optimal PGR concentrations is essential. However, PGR application needs to be optimized while keeping in mind the risk of induction of somaclonal variations. Indeed, somaclonal variations such as variegations and fruit set abnormalities in tissue-culture-produced date palm have already been reported [38]. Cytofluorimetric analyses revealed that the regeneration process under study did not induce any detectable change in ploïdy level and DNA content of regenerated plantlets. It must be noticed that more discrete changes in DNA such as aneuploidy cannot be detected using cytofluorimetry. Furthermore, an epigenetic origin for somaclonal variants has been proposed in oil palm [39] and date palm [38]. In oil palm, a correlation between somaclonal variation and the methylation status of genomic DNA has been established [39]. In this case, off-types could not be identified by techniques relaying on genomic DNA sequence analyses [40]. To limit the risks of producing somaclonal variants, PGR concentrations and applications need to be lowered and protocols need to be evaluated regarding clonal fidelity in the field. 

## Figures and Tables

**Table 1:** Influence of BAP on the biomass changes in suspension cultures and somatic embryos development after 5 weeks on PGR-free medium.

BA (mg/L)	Suspension culture Fresh weight (g)	Embryo number per Petri dish	Vitreous embryo number per Petri dish
0	1.37^c^	21^b^	1^e^
0.5	1.50^b^	29^a^	1.5^d^
1	1.63^ab^	18^c^	3^c^
1.5	1.66^ab^	14^d^	4.7^b^
2	1.77^ab^	11^e^	5.1^a^

Average values were calculated from 5 repetitions per condition of medium; letters indicate significative differences according to Newman and Keuls test at the level of 5%.

**Table 2:** Influence of NAA on rooting after 4 weeks.

NAA (mg/L)	Root number per plant	Root lengh (cm)
0	13^a^	1.4^b^
1	1^b^	4.9^a^

Average values were calculated from 5 repetitions per condition of medium; letters indicate significative differences according to Newman and Keuls test at the level of 5%.

**Table 3:** Quantification of nuclear DNA from cells of leaf tissue of seedlings and clones produced from cell suspensions in cultivars Ahmar and Amsekhsi.

Genotypes	qADN (pg)	Standard deviation (pg)	Number of repetitions
Ahmar clones	1,72	0,01	15
Ahmar seedlings	1,74	0,02	7
Amsekhsi clones	1,73	0,01	18
Amsekhsi seedlings	1,73	0,02	10
